# *In-vitro* evaluation of selected Egyptian traditional herbal medicines for treatment of alzheimer disease

**DOI:** 10.1186/1472-6882-13-121

**Published:** 2013-05-30

**Authors:** Shereen K Ali, Ahmed R Hamed, Maha M Soltan, Usama M Hegazy, Esameldin E Elgorashi, Ibrahim A El-Garf, Ahmed A Hussein

**Affiliations:** 1 Department of Phytochemistry, National Research Centre, 13211 Dokki, Cairo, Egypt; 2 Pharmaceutical Research Group, Centre of Excellence for Advanced Sciences, National Research Centre, 13211 Dokki, Cairo, Egypt; 3 Molecular Biology Department, Genetic Engineering and Biotechnology Division, National Research Centre, 13211 Dokki, Cairo, Egypt; 4 Phytomedicine Programme, Department of Paraclinical Sciences, Faculty of Veterinary Science, University of Pretoria, Onderstepoort 0110, Pretoria, South Africa; 5 Department of Botany, Faculty of Science, Cairo University, El-Giza, Egypt; 6 Chemistry Department, University of Western Cape, Private Bag X17, Belleville 7535, South Africa

**Keywords:** Egyptian herbal medicine, Unani medicine, Alzheimer’s disease, Anti-acetylcholinesterase, Anti-inflammatory, Anti-oxidant, *Adhatoda vasica*, *Ferula assafoetida*

## Abstract

**Background:**

Egyptians recognized the healing power of herbs and used them in their medicinal formulations. Nowadays, “Attarin” drug shops and the public use mainly the Unani medicinal system for treatment of their health problems including improvement of memory and old age related diseases. Numerous medicinal plants have been described in old literature of Arabic traditional medicine for treatment of Alzheimer’s disease (AD) (or to strengthen memory).

**Methods:**

In this study, some of these plants were evaluated against three different preliminary bioassays related to AD to explore the possible way of their bio-interaction. Twenty three selected plants were extracted with methanol and screened *in vitro* against acetylcholinesterase (AChE) and cycloxygenase-1 (COX-1) enzymes. In addition, anti-oxidant activity using DPPH was determined.

**Results:**

Of the tested plant extracts; *Adhatoda vasica* and *Peganum harmala* showed inhibitory effect on AChE at IC_50_ 294 μg/ml and 68 μg/ml respectively. Moreover, *A. vasica* interacted reversibly with the enzyme while *P. harmala* showed irreversible inhibition. *Ferula assafoetida* (IC_50_ 3.2 μg/ml), *Syzygium aromaticum* (34.9 μg/ml) and *Zingiber officinalis* (33.6 μg/ml) showed activity against COX-1 enzyme. Potent radical scavenging activity was demonstrated by three plant extracts *Terminalia chebula* (EC_50_ 2.2 μg/ml), *T. arjuna* (3.1 μg/ml) and *Emblica officinalis* (6.3 μg/ml)*.*

**Conclusion:**

Interestingly, differential results have been obtained which indicate the variability of the mode of actions for the selected plants. Additionally, the reversible interaction of *A. vasica* against AChE and the potent activity of *F. assafoetida* against COX-1 make them effective, new and promising agents for treatment of AD in the future, either as total extracts or their single bioactive constituents.

## Background

Alzheimer’s disease (AD) is a neurodegenerative disorder characterized by a progressive decline of memory and cognition. Amyloid-β (Ab), neurofibrillary tangles (NFT) and synaptic loss; particularly, the deficiency of acetylcholine (ACh) and the degeneration of cholinergic neurons in the cortex and hippocampus, nucleus basalis of Meynert, are the hallmarks of AD [1,2]. A loss of ACh is considered to play a vital role in the learning and memory deterioration of AD patients. Acetylcholine is an organic molecule liberated at nerve endings as a neurotransmitter. It is produced by the synthetic enzyme choline acetyltransferase which uses acetyl coenzyme-A and choline as substrates for the formation of acetylcholine in specific cells known as cholinergic neurons. Neurotransmitter disturbances and insufficient cholinergic functions are identified among the pathological features in central nervous system disorders [3].

Because of the complication of ACh deficiency in AD patients, elevating ACh level is an essential target for treatment. There are many strategies that can be used to enhance ACh level such as using ACh precursor (choline) [4], muscarinic and nicotinic agonists [5], ACh releasers [6] and AChE inhibitors [7]. However AChE inhibitors have some complications such as toxicity or resistance by increasing AChE expression level [8], but use of AChE inhibitors in AD patients has been the most effective strategy up to date.

Inflammation is a disorder involving localized increase in the number of leukocytes and a variety of complex mediator molecules. Prostaglandins are ubiquitous substances that initiate and modulate cell and tissue responses involved in inflammation. Their biosynthesis has also been implicated in the pathophysiology of cardiovascular diseases, cancer, colonic adenomas and Alzheimer’s disease [9].

Oxidative stress refers to the physiological condition at which the capacity of the endogenous antioxidant system fails to cope with the damaging effects of free radicals. Strong experimental evidences have been established about the oxidative stress theory of AD pathogenesis where oxidative damage plays a major role in neurological degeneration [10].

The ancient Egyptians had a system of medicine that was very advanced for its time and influenced later medical traditions. When the Arabs came to Egypt, Arabic medicine was practiced and the art of healing made use of all available knowledge gained from different civilizations such as the Persian, Chinese, Greek, as well as the Ancient Egyptian. The books written by some famous scholars such as Al-antaki [11], Al-turkimany [12], Ibn Sina [13], and Ibn el-Bitar [14] which represent the main references in herbal shops (known as Attarin), described in their books a number of conditions related to AD and recommended numerous herbal medicine to improve the old ageing problems including AD. In this study we analysed these books and abstracted the information on plants described for the treatment of old age diseases like Alzheimer, joint inflammations …etc.

Currently, a few drugs are available in the market as safe and effective for the treatment of AD. Thus, in this article we evaluated *in vitro* some Egyptian herbal medicines that have been highly recommended in old Arabic literature for treatment of AD to discover *newly* potent and safe natural therapeutic agents for treatment of AD.

## Methods

### Plant materials

Selection of plant species screened in this study was based on their uses in Egyptian traditional medicine (Table 1). Information was gleaned from different sources of old Arabic literature available which are believed to be the main references used in the “Attarin” shops in Cairo. In this study we reviewed the information given by some scholars like Dawood el Antaki [11], Al-torkmany [12], Ibn Sina [13], and Ibn el-Bitar [14].

**Table 1:** Egyptian herbal medicines reported for treatment of age- related diseases

**No.**	**Plant species**	**Plant family**	**Voucher No.**	**Part used**	**Collection site**	**Arabic name**	**Traditional uses**
1	*Adhatoda vasica* Nees.	Acanthaceae	STDF-1	Aerial parts	El-Orman garden		No traditional use reported
2	*Aloe vera.* L.	Aloaceae	STDF-2	Dried juice	El-Orman garden		Improves mental capacity, benefits vitality, anti-depressant [12]. Used by pharaohs for chest pains, headaches, skin diseases and allergies [21],
3	*Anacyclus pyrethrum* L*.*	Asteraceae	STDF-5	Roots	Herbal shop		Nerve tonic, improves cerebral blood circulation, remedy for paralysis [12].
4	*Boswellia sacra* Flueck.	Burseraceae	STDF-6	Gum	Oman		Anti-depressant enhances mental capacity, cures frequent forgetfulness, [12,14]. *Boswellia* sp. was used by ancient Egyptians for rheumatism, joint pain and facial wrinkles [21].
5	*Brassica rapa ssp rapa*	Brassicaceae	STDF-8	Root	Local market		Aphrodisiac, anti-ageing, hearing disorders [12].
6	*Brassica nigra* L*.*	Brassicaceae	STDF-9	Seeds	Herbal shop		Anti-ageing strengthens the vitality [21]. Aphrodisiac, joints disorders and chest pain [11,13,14]. The Pharaohs used mustard seeds to treat muscle, joint and chest pains [21].
7	*Emblica officinalis* Gaertn	Euphorbiaceae	STDF-10	Fruits	El-Fayom		Improves memory, stimulant, and restoratives for all organs [21].
8	*Ferula assafoetida* Boiss. & Buhse	Apiaceae	STDF-12	Gum	Iran		Stimulant, strong aphrodisiac, strong nerve tonic, relieves on-going mental and physical fatigue, joints inflammation, depression and sadness [12]. Treat weakness of sexual desire and nerves [11].
9	*Melilotus officinalis* (L.) Pall.	Fabaceae	STDF-13	Aerial parts	Orman garden		Joints pains [12].
10	*Cassia fistula* L*.*	Fabaceae	STDF-14	Fruits	Orman garden		Tonic, detoxicant [12]. expectorant for brain and chest problems [11]. Relieves inflammations of nerves and joints [13,14].
11	*Nerium oleander* L*.*	Apocynaceae	STDF-15	Leaves	Orman garden		Highly toxic, relieves knee and back pain [12].
12	*Nigella sativa* L*.*	Ranunculaceae	STDF-16	Seeds	Herbal shop		Stimulant, improving memory, resolutive, considered as an adaptogen [12].
13	*Peganum harmala* L*.*	Zygophyllaceae	STDF-21	Seeds	South Sinai		Hallucinogenic, epilepsy, mental and nervous illnesses, relieves joints inflammation [12]. Cures headaches, strokes, numbness, epilepsy and forgetfulness [11].
14	*Piper nigrum* L*.*	Piperaceae	STDF-22	Seeds	Herbal shop		Stimulant, memory enhancer, sharpens the mind, and for strokes [11,12]. *Piper* sp. (*Piper cubeba*) used by Pharaohs against different types of infections and headaches [21].
15	*Rheum palmatum* L*.*	Polygonaceae	STDF-23	Stem	Herbal shop		Anti-ageing, for dyspepsia, improves memory, and maintains healthy mind [12].
16	*Rosmarinus officinalis* L*.*	Lamiaceae	STDF-24	Aerial parts	Orman garden		Sharpens the mind, anti-depressant, anxiety, poor memory, and rheumatoid arthritis.
17	*Ruta graveolens* L*.*	Rutaceae	STDF-25	Leaves	Local market		Memory enhancer, relieves strokes, tremors, convulsion and epilepsy, joint pains [12].
18	*Salvia triloba* L*.*	Lamiaceae	STDF-26	Aerial parts	Sinai (El-A’rish)		Anti-inflammatory, nerve tonic, and memory enhancer.
19	*Syzygium aromaticum* (L.) Merrill & Perry	Myrtaceae	STDF-27	Pud	Herbal shop		General tonic and memory enhancer [12]. Stimulant for brain, and anti-depressant [11].
20	*Terminalia arjuna* (Roxb.) Wight & Arn.	Combretaceae	STDF-28	Fruits	El-Fayom		Highly recommended for ageing diseases, improves memory and brain function, keeps the brain young and healthy [12]. Strengthens the senses and the brain, improves memory [11,13].
21	*Terminalia chebula* Retz*.*	Combretaceae	STDF-29	Fruits	El-Giza		Traditionally used like *T. arjuna*.
22	*Teucrium polium* L*.*	Lamiaceae	STDF-30	Aerial parts	South Sinai		Improves mental performance, and concentration [12].
23	*Zingiber officinale* Roscoe	Zingiberaceae	STDF-31	Rhizome	Local market (Mepaco)		Memory enhancer, for joints inflammation [11-13].

Plant materials (leaves, roots and seeds) were collected from either their natural habitats or the local market (Table 1). Two plants *Boswellia scara* (supplied and identified by Dr. A. al_Adawi, Ghadafan Agriculture Research Station, Ministry of Agriculture and Fisheries, Sohar, Sultanate of Oman), and *Ferula assafoetida* (supplied by Dr. M. Ziaratnia, Research Institute of Food Science and Technology, Isfahan, Iran). Voucher specimens (Table 1) were identified by Prof. Ibrahim El-garf, a co-author of this article, and deposited in the Department of Phytochemistry, National Research Centre, Egypt. The collected fresh materials were dried, powdered and extracted by homogenization with methanol (10 ml g^−1^), using electrical blender and macerated overnight then filtrated, the residues were re-extracted three times with fresh solvent. The filtrates were combined and the solvent removed at 45°C under reduced pressure. The total extracts were kept at ~ −5°C for further use.

### The multi-well plate AChE inhibition assay

The AChE inhibitory activity of each extract was tested using 96 well micro-plate assay based on previously published methods [15,16] with minor modifications. Each extract (25 μl of 10× of final concentrations in DMSO) was dispensed in duplicates onto 96 well micro-plate and mixed with 200 μl of Ellman’s mixture containing 10 mM Tris–HCl, pH 8, 0.1% bovine serum albumin (BSA, fraction V), 1.5 mM acetylthiocholine iodide (ATCI, Sigma-Aldrich, Germany) and 3 mM 5,5'-dithio-bis-(2-nitrobenzoic acid) (DTNB, Sigma-Aldrich, Germany). The control wells contained the extract vehicle (DMSO) instead of the extract. The reaction was started with the addition of enzyme solution (25 μl, 0.1 U/ml). Autohydrolysis of the substrate was corrected by replacing the enzyme with 25 μl of enzyme buffer (10 mM Tris–HCl, pH 8, containing 0.1% BSA) in duplicate wells. The enzymatic activity was monitored kinetically at 450 nm every 30 s intervals for 3 min at 30°C (linear reaction). The enzyme rate was calculated from the slope of the curve of absorbance change vs time. As screening strategy, final concentration of 1000 μg/ml from each extract was examined and the average % inhibition was calculated relative to the enzyme rate at the vehicle control wells according to equation 1:

$$\%\;\mathrm{Inhibition}=\times \left[\frac{\mathrm{mean}\;\mathrm{slopes}\;\mathrm{of}\;\mathrm{the}\;\mathrm{vehicle}\;\mathrm{control}-\mathrm{mean}\;\mathrm{slopes}\;\mathrm{of}\;\mathrm{the}\;\mathrm{sample}}{\mathrm{mean}\;\mathrm{slopes}\;\mathrm{of}\;\mathrm{the}\;\mathrm{vehicle}\;\mathrm{control}}\right]$$

Equation 1. Calculation of the average % inhibition of different extracts on AChE.

Five serial dilutions were prepared from the extracts that showed more than 50% inhibition to determine the IC_50_ (extract concentration producing 50% inhibition of AChE activity as generated by non-linear regression analysis). Galanthamine (Sigma-Aldrich, Germany) served as positive control.

### Determination of the inhibition type of plant extracts on AChE

The type of inhibition of AChE by *P. harmala* and *A. vasica* extracts (reversible or irreversible inhibition) was determined by measuring the restored AChE activity by 10 time dilution of plant extract concentration after mixing and incubation of AChE and plant extract. AChE activity was measured after gentle mixing of 110 μl of (100 μl enzyme:10 μl plant extract) with 890 μl of mixture containing 10 mM Tris–HCl, pH 8, 0.1% BSA, 1.5 mM ATCI, 3 mM DTNB and 90 μl plant extract. In a separate experiment, the dilution effect of plant extract on AChE activity was measured after gentle mixing 110 μl of (100 μl enzyme:10 μl plant extract) with 890 μl of the same above mixture except that 90 μl plant extract was replaced with 90 μl DMSO (solvent). In reversible inhibition, AChE activity can be restored by dilution of plant extract, while there is no change in AChE activity with dilution of plant extract in irreversible inhibition.

### Cyclooxygenase-1 assay

Inhibition of prostaglandin biosynthesis by the plant extracts was investigated using COX-1 assay [17]. Indomethacin was included as a standard. Per cent inhibition of plant extracts was calculated by comparing the amount of radioactivity present in the sample with that in the solvent blank.

### Antioxidant activity: 2,2-diphenyl-1-picrylhydrazyl (DPPH) radical scavenging assay

Plant extracts/compounds were prepared in DMSO as 10× stocks from each test concentration (between 0–100 μg/ml) and briefly sonicated when necessary in an ultrasonic water bath. Plant extracts/compounds producing radical scavenging activities equal to or higher than 50% at 100 μg/ml in a preliminary screen were further tested and EC_50_ (concentration of the extract/compound producing 50% scavenging of DPPH radicals) determined using non-linear regression analysis of the dose-%AA relationship (Equation 1). Three reference radical scavengers (quercetin, gallic acid and *t*-butylhydroquinone) were tested in the assay as positive controls. The assay method used in the present study was based on a modified procedure [18] which is based essentially on previously published literature [19]. The plant extract/compound stock solutions (20 μl/well) were dispensed in duplicates onto 96-well plates (flat-bottomed, Greiner bio one, Belgium). The assay was started with the addition of DPPH reagent (0.004% wt/v in methanol, 180 μl/well). Appropriate blanks were prepared using the solvent only in addition to the same amount of DPPH reagent to get rid of any inherent solvent activity. Negative controls were also run in parallel to correct for any non-DPPH absorbance by coloured extracts at the test wavelength. The plate was immediately shaken for 30 seconds and incubated in the dark for 30 minutes at room temperature. The remaining DPPH was measured in the microplate reader at 540 nm. The percentage of antioxidant activity (%*AA)* was calculated according to equation 2:

$$\%\;\mathrm{Antioxidant}\;\mathrm{activity}\;\mathrm{DPPH}\;\left(\mathrm{\%AA}\right)=100\times \frac{\left[\mathrm{OD}540\left(\mathrm{blank}\right)-\mathrm{OD}540\;\left(\mathrm{sample}\right)\right]}{\mathrm{OD}540\;\left(\mathrm{blank}\right)}$$

Equation 2. Calculation of the % AA for DPPH assay. OD_540_ (blank) and OD_540_ (sample) are the averages of duplicate determinations of the corrected readings of blank and sample at 540 nm, respectively.

## Results

The plants (Table 1) were selected based on their traditional uses for treatment of AD or age related diseases except for *A. vasica* which was selected on the basis of chemotaxonomy. The plants were collected from their natural habitats or from the “*Attarin*” or the herb shops. A small portion of plant parts (100 g) were extracted and tested in different *in-vitro* bioassays related to AD. The medicinal uses of the listed plants (Table 1) were discussed in detail by numerous scholars as Ibn Sina “Avicenna”, Ibn El Beitar, El Baironi, Al Antaki, Al Mo’tamed [11-14,20].

**Figure 1:**
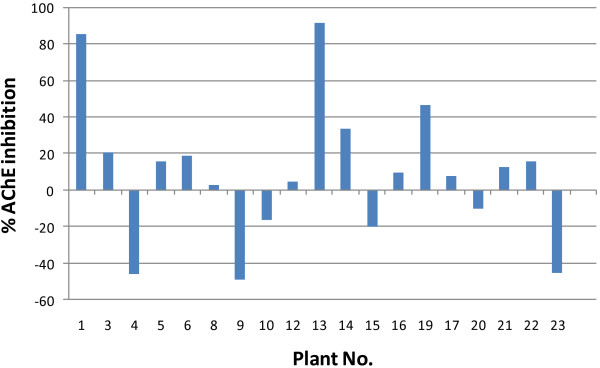
**% inhibition of AChE by different plant extracts at 1000 μg/ml.** Plant No. on x-axis refers to the corresponding numbered plants in Table 1.

The inhibition effect of the methanolic extracts from the 23 different extracts on AChE activity was screened (Table 2, Figure 1). The screening was performed at a concentration of 1000 μg/ml and the activity guidelines of our program only considered the extracts as active if they only inhibited the enzyme more than 50%. The screening showed different effects on AChE activity as shown in Table 2. Extracts from *M. officinalis*, *B. sacra*, and *Z. officinalis* activated AChE more than 45%. Only, two species namely *A. vasica* and *P. harmala* inhibited AChE by 86 and 90% respectively (Figure 1). Further testing and analyses of the inhibition of AChE by *A. vasica* and *P. harmala* revealed the IC_50_ values of 294 and 68 μg/ml respectively.

**Table 2:** Biological activities of Egyptian herbal medicines against different bioassays related to AD

**No.**	**Plant species**	**Screening data**			**IC**_**50**_**(μg/ml)**		
		**% inhibition of ACHE**^**a**^	**% inhibition of COX-1**^**b**^	**DPPH (%AA)**^**b**^	**ACHE**	**COX-1**	**DPPH**
1	*A.vasica*	86.0	-	19.0	294	N.D	>100
2	*A. vera*.	-	-	31.0	-	N.D	>100
3	*A. pyrethrum* L*.*	21.0	-	90.0	N.D	N.D	26.3
4	*B. sacra*	−46.0	15.1 ± 5.7	8.0	N.D	N.D	>100
5	*B. alba*	16.0	52.3 ± 2.1	24.0	N.D	N.D	>100
6	*B. nigra.*	19.0	10.9 ± 1.6	42.0	N.D	N.D	>100
7	*E. officinalis*	-	-	97.0	N.D	N.D	6.3
8	*F. assafoetida*	3.0	96.7 ± 1.3	17.0	N.D	3.2	>100
9	*M. officinalis*	−49.0	-	6.0	N.D	N.D	>100
10	*C. fistula.*	−16.0	19.2 ± 4.8	66.0	N.D	N.D	75.0
11	*N. oleander.*	-	-	60.0	N.D	N.D	64.5
12	*N. sativa.*	5.0	55.4 ± 8.8	19.0	N.D	N.D	>100
13	*P. harmala.*	92.0	-	41.0	68	N.D	>100
14	*P. nigrum.*	34.0	-	30.0	N.D	N.D	>100
15	*R. palmatum.*	−20.0	-	95.0	N.D	N.D	14.2
16	*R. officinalis.*	10.0	11 ± 5.4	82.0	N.D	N.D	19.4
17	*R. graveolens.*	8.0	-	69.0	N.D	N.D	61.0
18	*S. triloba.*	-	-	93.0	N.D	N.D	20.7
19	*S. aromaticum*	47.0	80.3 ± 0.9	89.0	N.D	34.9	15.9
20	*T. arjuna*	−10.0	-	96.0	N.D	N.D	3.1
21	*T. chebula*	13.0	-	95.0	N.D	N.D	2.2
22	*T. polium*	16.0	-	51.0	N.D	N.D	96.4
23	*Z. officinalis*	−45.0	68.2 ± 3.1	47.0	N.D	33.6	>100
	Galanthamine	n/a	n/a	n/a	9.4	n/a	n/a
	Indomethancin	n/a	n/a	n/a	n/a	0.61	n/a
	Reference DPPH scavengers:						
	t-BHQ						2.8
	Gallic acid						1.2
	Quercetin						4.5

^a^at 1000 μg/ml, ^b^at 100 μg/ml, *N.D* not detected, *n/a* not applicable.

The inhibition type of *A. vasica* and *P. harmala* was determined by assaying the change in the remaining AChE activity of the mixture of AChE and the plant extract before and after the dilution of the plant extract in the same mixture, while, AChE activity increased 5 fold by 10 times dilution of *A. vasica*, the same dilution of *P. harmala* did not show any effect on the remaining activity of AChE after dilution. This result indicates that AChE is inhibited reversibly by *A. vasica* and irreversibly by *P. harmala.*

Screening of the extracts against COX-1 enzyme at 100 μg/ml showed that six extracts demonstrated more than 50% inhibition (Table 2). The IC_50_ of the active extract showed potent inhibitory activity for *F. assafoetida* (IC_50_ 3.2 μg/ml) and moderate activity for *Z. officinalis* (33.6 μg/ml).

Table 2 shows the anti-oxidant results of the tested plant extracts, three of them; *T. chebula* (EC_50_ 2.2 μg/ml), *T. arjuna* (3.1 μg/ml) and *E. officinalis* (6.3 μg/ml) were particularly strong antioxidants when compared to the reference radical scavengers (*t*-BHQ, gallic acid and quercetin) recording EC_50_’s < 10 μg/ml. Five species showed the activity at EC_50_’s of 10–30 μg/ml; these were *R. palmatum* (EC_50_ 14.2 μg/ml), *S. aromaticum* (15.9 μg/ml), *R. officinalis* (19.4 μg/ml), *S. tribula* (20.7 μg/ml) and *A. pyrethrum* (26.3 μg/ml). Another four species *R. graveolens*, *N. oleander*, *C. fistula* and *T. polium* showed the activity at EC_50_ of 30–100 μg/ml. The rest of the examined plant species either showed weak activity or were inactive at 100 μg/ml.

## Discussion

Ancient Egyptians were familiar with drug preparation from plants and herbs such as cumin, fennel, caraway, aloe, safflower, pomegranates, and castor and linseed oils [21]. However, nowadays, the majority of the herbal medicine information is coming from Unani medicine, some of the plant still originated from pharaonic era, and still used for treatment of different diseases like *Boswellia sp.,* aloe, and mustard.

The deficiency of ACh is one of characteristics of AD and responsible for most of the AD symptoms such as decline of memory and cognition of the AD’s patients. AChE inhibitors such as tacrine, donepezil, rivastigmine, and galanthamine are effective anti-AD drugs in the market [22]. The side effects of anti-AChE drugs such as toxicity, tolerability, and loss of efficiency stimulates the researchers to screen alternative natural anti-AD drugs for medication switch [23].

In the present work, the selected extracts were screened for AChE inhibition. Only *A. vasica* and *P. harmala* showed inhibitory activity against AChE with IC_50_ value 294 and 68 μg/ml, respectively. Both plants contain β-carboline alkaloids, which demonstrated potent activity against AChE [24]. Extracts from natural resources usually containing un-determined number of secondary metabolites and expected to play different role upon their interaction with human biological system. In this study the major biological activity demonstrated by both extracts could be attributed to the dominant major constituents in each extracts which should be able to go through blood brain barrier and interact with the active sites. The major constituent of *P. harmala* is harmaline and *A. vescia* contains vasicine (from 0.0541 to 1.105%). Generally β-carbolines are a large group of natural indole alkaloids that are widely distributed in nature. They possess diverse pharmacological activities such as sedative, hypnotic, anxiolytic, anticonvulsant, antitumor, antithrombotic, antiparasitic, antimicrobial, as well as antiviral activities [24]. Harmaline, the major active constituent of *P. harmala* is a common dihydro β-carboline type, it possess interesting pharmacological activities and can interact with several enzymes and neurotransmittors including topoisomerase I, and monoamine oxidase-A [25,26]. The different activities demonstrated by both major compounds could explain the difference in relative potential IC_50_ values of both plants. Although, *P. harmala* has been used in traditional medicine, there are reports of severe intoxication in cattle, donkeys, sheep and horses [27]. Digestive and nervous syndromes have been reported in animals that consume a sub-lethal amount of the plant. Harmaline and harmine are toxic alkaloids characterized in the seeds of *P. harmala*. Harmaline is almost twice as toxic as harmine and in moderate doses cause tremors and clonic convulsions, but with no increase in spinal reflex excitability [28]. *A vasica* showed safety when intragastrically administrated at 2.5 g/kg, clinical trials performed on combination preparations containing *A*. v*asica* showed no serious adverse effects [29].

Although IC_50_ value of *P. harmala* is about four times higher than that of *A. vasica*, the inhibition type study showed that *A. vasica* reversibly inhibits AChE and can be used for AD’s medication rather than *P. harmala* which inhibits irreversibly AChE. This recommendation was supported by the toxicity reports in literature which indicated the higher safety margin of *A. vasica* as compared to *P. harmala*.

According to the best of our knowledge this is the first report about the reversible anti-chloinsterase interaction of *A vasica* extracts growing in Egypt, which add new value and activity to this important plant.

Anti-inflammatory COX-inhibiting NSAIDs have received increased attention in experimental and therapeutic trials for Alzheimer’s disease. Interestingly, COX-1-expressing microglia surrounds amyloid plaques. There is no evidence that COX-1 expression in microglia is changed in AD brain. However, accumulation of COX-1-expressing microglia in AD could result in local increase in prostaglandin synthesis and oxidative stress. *F. assafeotida* demonstrated potent inhibitory activity against COX-1 enzyme. Asafoetida used in traditional medicine to improve memory and as an antihelminthic, antispasmodic and antibacterial agent [11-14]. *Z. officinalis* showed potent inhibitory activity against COX-1 enzyme, and also, demonstrated high radical scavenging properties, which may attributed to their contents of gingerols and shoagols. Further understanding of the role of COX inhibitory activity of herbal medicine in mechanisms leading to AD generation is critical to the future development of NSAID therapy for AD from traditional medicine [30].

Increased oxidative stress causes cell damage in the form of protein, lipid, and DNA oxidations. Elevated ROS levels are also associated with increased deposition of amyloid- and formation of senile plaques, a hallmark of the AD brain. If enhanced ROS exceeds the basal level of cellular protective mechanisms, oxidative damage and cell death will result. Therefore, the plant extracts which demonstrated potent free radical scavenging properties particularly those showed EC_50_ < 10 μg/ml (*T. chebula, T. arjuna* and *E. officinalis*) expected to play a vital role in reducing the oxidative stress and this may explain their use in traditional medicine for improvement of AD and/or ageing related diseases.

*Brassica* was reported to be used traditionally against many human diseases including AD. It contains potential bioactive phytochemicals. Isothiocyanate derivatives from Brassicaceae increased NGF-induced neurite elongation by ∼ 70%. It’s also induced sustained production of β-tubulin in the presence of NGF enhancers [31]. Plant sterols including brassicasterol are solely dietary-derivable sterols that are structurally very similar to cholesterol and can cross the blood–brain barrier and accumulate within mammalian brain and may play an important role in protection against AD [32]. Sinapic acid showed anti-inflammatory and neuroprotective activities, the mechanism of action involve amelioration of Aβ(1–42) protein-related pathology including neuronal cell death and cognitive dysfunction [33]. Sinapine is another compound which is widely spread in Brasicaceae, significantly inhibited AChE activity on cerebral homogenate (IC_50_ 3.66 μmol/L^-1^) [34].

*Cassia fistula* native to southern Asia, and widely distributed in Egypt as an ornamental tree. The seeds from the fruit are well known in Unani traditional medicine and widely used for medicinal purposes. It was described as safe and efficient purgative even for pregnant women and for children. Recently, the effects of the seed extracts against ageing diseases have been documented. The ethanolic extract of the seeds of *C. obtusifolia* (synonym *C. fistula*) (COE), significantly attenuates memory impairment induced by scopolamine via acetylcholinesterase inhibition [35]. COE attenuated secondary Ca^2+^ dysregulation induced by NMDA (700 μM), while a pre-application of COE reduced NMDA-induced cell death. Furthermore, COE was neuroprotective against the mitochondrial toxin 3-NP (1 mM) [36]. Some of the isolated compounds were shown to inhibit the activities of β-secretase and enhance the memory in the animals with scopolamine-induced memory loss [37].

*Emblica officinalis* (Amla) grows in tropical and subtropical parts of East Asia, and was cultivated in Egypt in the last few years for its economic value. In traditional medicine, *E. officinalis* is used for various conditions like diarrhea, jaundice, inflammation, cerebral insufficiency and mental disorders [38]. It is used as a tonic for heart and brain in Unani medicine. The extract demonstrated various pharmacological activities. Amla churna (powdered dry fruit of amla) has also been reported to produce a dose-dependent improvement in memory scores of young and aged rats [39,40]. *E. officinalis* extract has an ability to improve or ameliorate spatial long-term memory and short-term memory attributable to mechanisms like antioxidant, anti-inflammatory, AChE inhibitory, hypolipidemic and neuroprotective activities [41].

*Nerium oleander* (oleander) belongs to the family Apocynaceae. It is widely cultivated as a garden plant, which showed interesting anticancer activity. Unani system recommended the topical uses of the plant more than the internal use, which should be administrated under supervision and with caution. The anti-ageing properties of the plant extract was documented recently, the polysaccharides isolated from the flowers of oleander showed potential neuroprotective activity against neuronal death in Alzheimer’s disease and the neuroprotective mechanism may primarily rely on inactivation of JNK signaling pathway [42,43]. Also, cardiac glycoside derivatives are proposed as treatment for Alzheimer’s disease, Huntington’s disease or stroke [44].

*Nigella sativa* is considered as an adaprogenic herb and is widely used in Egypt and other Arabic countries; it showed no activity *in vitro* against cholinesterase [45], but *in vivo,* the fixed oil has demonstrated noticeable spatial cognitive preservation in rats challenged with chronic cerebral hypoperfusion which indicates a promising prospective neuroprotective effect [46].

*Ruta graveolens* (common rue) is cultivated in many parts of the world; it has been used for centuries as a medical preparation. In Unani system it is used as stimulant, emmenagogue, diuretic, abortefacient, resolvent and brain tonic [47]. Methanolic and hexane extracts of *R. graveolens* showed potent inhibition of AChE and butyryl cholinesterase (BuChE) *in-vitro*[48]. Rue contains rutin, which, widely used as a drug to improve blood circulation and expected to contribute for such activities.

Sage is a common name for *Salvia* species, and highly appreciated over all the world, it is used for treatment of many diseases and also proved to have strong activity against AD, in Egypt *S. triloba* is called Maramaria and it is used as condiment and tea. The plant has been reported in old Arabic literature to improve the mental power [49,50].

Black pepper (*Piper nigrum*) is a flowering vine in the family Piperaceae [51]. The plant has been used effectively for the treatment of AD. Piperine is a major plant alkaloid present in black pepper (*Piper nigrum*) and long pepper (*Piper longum*), which are among the most common spices consumed by a large number of people worldwide. This plant is known to possess several pharmacological actions, such as antimicrobial, antifungal, anti-inflammatory and antioxidant effects [52]. Piperine demonstrated in *in vitro* studies to protect against oxidative damage by inhibiting or quenching free radicals and ROS, lower lipid peroxidation *in vivo* and beneficially influence cellular thiol status, antioxidant molecules and antioxidant enzymes in a number of experimental situations of oxidative stress [53].

A recent *in vivo* work conducted by our group, revealed a significant reduction of the oxidative stress status and amelioration of the neurodegeneration characteristic of Alzheimer’s diseases in rats using *P. nigrum* and *S. triloba*. It is noteworthy that *S. triloba* extract showed more interest in improvement of AD in rats [54].

*Terminalia* species belong to the family combretaceae. They are extensively used in Unani, Ayurveda and homeopathic medicine. *T. chebula* is a popular traditional medicine in many countries including Egypt. It has a wide spectrum of pharmacological activities and reported as antioxidant, antidiabetic, antibacterial, antiviral, antifungal …etc. According to Unani medicine, emulsifying of one fruit every day prevents ageing and keeps the person very healthy. Recent literature supported the anti-ageing properties of *Terminalia* species. Phenolic constituents from *T. chebula* showed strong AChE and BChE inhibitory activities, and antioxidant activity [55]. *T. chebula* has been recommended for old age diseases [56]. Oral administration of different doses of aqueous extract of *T. arjuna* causes significant elevation in activities of catalase, superoxide dismutase and glutathione *S* transferase. Also, *T. arjuna* is found to down regulate anaerobic metabolites by inhibiting the activity of lactate dehydrogenase in lymphoma bearing mice. The strong antioxidant action of aqueous extract of *T. arjuna* may play a role in treatment of age-related diseases such as cancer and coronary heart disease and neurodegenerative disorders [57].

The examined biological properties; anti-AChE, anti-oxidant and anti-inflammatory of the selected species revealed a diversity of the active species suggesting a different mechanisms. Additionally, animal based *in vivo* research during the last ten years revealed interesting activities for the majority of the plants listed in Table 1 as discussed above, which, justify their use in traditional medicine to improve memory or treatment of ageing diseases including AD by traditional practitioner overall the world including Egypt.

## Conclusion

The reputed medicinal properties of plants have been documented for centuries in different cultures including Egypt, and there are many plant species that have been traditionally used for memory disorders as listed in Table 1. Different results have been obtained which indicate the variability of the mode of actions for the selected plants. Additionally, the reversible interaction of *A. vasica* against AChE and the potent activity *F. assafoetida* against COX-1 making them effective, new and promising agents for treatment of AD in the future, either as total extracts or their single bioactive constituents.

## Competing interests

The authors declare that they have no competing interest.

## Authors’ contributions

SKA and AAH carried out the collection and extraction of plant materials and drafting the manuscript. ARH, MMS, UMH participated in acetylcholinesterase, DPPH, inhibition type bioassays, analysis and interpretation of data and in the drafted the manuscript. EEE performed COX-1 bioassay. IAE-G identified the plant materials. All authors read and approved the final manuscript.

## Pre-publication history

The pre-publication history for this paper can be accessed here:

http://www.biomedcentral.com/1472-6882/13/121/prepub
